# Challenges of Recruiting Caregiver and Care Receiver Dyads for a Randomized Clinical Trial

**DOI:** 10.1093/geroni/igaa057.1147

**Published:** 2020-12-16

**Authors:** Christine Fruhauf, Arlene Schmid, Neha Prabhu, Laura Swink, Jennifer Portz, Heather Leach, Mary Hidde, Marieke Van Puymbroeck

**Affiliations:** 1 Colorado State University, Fort Collins, Colorado, United States; 2 Colorado State University, Fort Collinc, Colorado, United States; 3 UCHealth Anschutz, Denver, Colorado, United States; 4 University of Colorado Anschutz, Aurora, Colorado, United States; 5 Clemson University, Clemson, South Carolina, United States

## Abstract

The recruitment of participants for chronic disease clinical trial research is often challenging. Further complicating participant recruitment occurs when the intervention is dyadic (i.e., simultaneously includes both care receivers and caregivers or recruiting pairs of people). Despite the strong support in favor of dyadic interventions for certain chronic diseases (e.g., among cancer and stroke survivors and their caregivers), researchers have not systematically shared challenges and opportunities for dyadic recruitment. During the recruitment for a yoga and self-management education intervention for people with chronic pain and their caregivers, several steps were taken to recruit and screen potential participants for the study. In this presentation, we will provide an overview of common recruitment challenges for physical activity and chronic disease self-management studies as well as the actual challenges encountered and our procedures for overcoming these obstacles. We will present our consort figure with attention toward inclusion and exclusion criteria of both care receivers and caregivers. Additional discussion will include specific challenges encountered when recruiting and screening caregivers (i.e., after the care receiver has been screened). The need for innovative clinical trial research with caregivers and care recipient dyads is essential as new care practices continue to evolve and demands on health care utilization increase. Lessons learned from this study may prove useful for future researchers as they embark on developing and testing dyadic interventions among adults with chronic disease and their caregivers.

